# “Context, Please?” The Effects of Appearance- and Health-Frames and Media Context on Body-Related Outcomes

**DOI:** 10.3389/fpubh.2021.637354

**Published:** 2021-07-30

**Authors:** Alice Binder, Selina Noetzel, Ines Spielvogel, Jörg Matthes

**Affiliations:** Department of Communication, Faculty of Social Sciences, University of Vienna, Vienna, Austria

**Keywords:** framing, social media, traditional media, mood, body satisfaction, social comparison

## Abstract

Promoting health-related behaviors such as healthy eating or doing sports are important to counteract the problem of obesity, which is on the rise. In this regard, initial studies suggest that appearance compared to health framing can lead to negative body-related outcomes in young women. This study aimed to extend these findings by investigating the role of the context. Furthermore, as previous studies focused on young women only, we considered a more diverse sample. This seems especially important as health campaigns focusing on healthy eating and sports should appeal to a more diverse population. This experimental study (*N* = 286) follows a 2 (appearance frame vs. health frame) × 2 (social media vs. magazine website) between-subjects design. Results revealed that exposure to appearance-focused framing led to a lower positive mood compared with exposure to health-focused framing. These effects were most prevalent in overweight and obese participants. Moreover, participants in the social media condition showed lower body satisfaction and lower positive mood compared with participants in the magazine website condition independent of the frame. No other interaction effects occured. Overall, health promoters should focus their campaigns on the health aspects of health-related behaviors and should consider promotion on social media platforms. Also, they should keep in mind that not only the framing, but also the context, might have effects on body-related outcomes.

## Introduction

The media has the ability to shape the awareness of people on what behaviors are important in order to reach a specific goal. In this regard, health messages promoting healthy eating or doing sports might act as crucial channels in combating the public health threats on rise worldwide, such as overweight and obesity (1). Reducing overweight and obesity is crucial for public health, as they are known as risk factors for several cardiovascular diseases and also some types of cancer (2).

However, current research revealed that, in different contexts (i.e., magazines, social media), such health behaviors (i.e., engaging in physical activity, maintaining a healthy diet) are often portrayed in connection to the goal of improving the appearance of an individual, whereas health benefits are not emphasized (3–6). This seems problematic because the reasoning for engaging in health-related behaviors has been shown to be decisive (7). Hence, engaging in health-related behaviors in order to improve the appearance of an individual was associated with lower levels of self-esteem (7) and other negative psychological effects, such as body dissatisfaction or negative mood (7–9).

This might be particularly prevalent for presenting appearance-focused health content on social media, as this content is generally perceived as more realistic and is therefore probably more influential (10, 11). While one study already showed that appearance framing in magazine headlines compared with health framing in magazine headlines led to higher body shaming in young women (4), the effects of framing in different media contexts have not been investigated yet. Furthermore, most studies in this area of research focused only on young women (12). However, studies revealed that body-related concerns appear in women as well as in men and also across different age groups (13). Therefore, research should focus on more diverse samples to gain insights into whether the results found for young women also account for the general population. Particularly, this study tries to support health promoters with regard to their campaign strategies because—as overweight and obesity are increasing among all age groups and genders—their target group is the general population (1). Thus, insights into how the general population perceives and reacts to different cues in the promotion of health-related behaviors are crucial for implementing the right form of communication that benefits, but does not harm, its receivers.

In this study, we aimed to examine whether possible negative and positive outcomes of promoting health behaviors are driven by (1) the frame, that is, focusing on appearance-related or health-related aspects, (2) the context in which the content is presented (social media vs. magazine website), and (3) by the interaction of both. To the best of our knowledge, it is still unclear from the current scholarship if the context might moderate some of the negative effects of specific framing strategies. We test these effects on three outcomes: positive and negative mood as well as the body satisfaction of individuals. We also examine whether these effects are moderated by the body mass index (BMI) of the participants. Furthermore, we aimed to contribute to this field of research with a balanced sample in terms of gender as previous studies primarily neglected the effects on men. This seems important because health promoters could apply this knowledge to design more effective health campaigns and to gain some insights as to which media contexts might (not) be beneficial when promoting health messages.

### Framing Effects in Connection With Health Behaviors

The *Framing Theory* (14) describes the process of applying different strategies to emphasize certain aspects of a message while leaving others in the background. Thus, to *frame* is defined as to present “some aspects of a perceived reality and make them more salient in a communicating text” (p. 52) (15). However, different media frames might influence how people “come to define a problem or story for themselves (i.e., individual frames)” (p. 52) (4). Thus, highlighting certain factors related to health-related behaviors might lead to different interpretations of the same behaviors.

Specifically, studies in the research area of health communication often distinguish between two media frames: appearance frames and health frames. As content analyses showed (4, 5, 16, 17), appearance frames emphasize the importance of healthy eating and doing sports to look good (i.e., sexy, attractive), whereas health frames focus on the health aspects connected with such behaviors (i.e., healthy, feeling good). Overall, content analyses revealed that, in health and beauty magazines (4, 5, 16, 17), as well as on social media (3, 6, 18–20), health behaviors are oftentimes promoted in connection with appearance aspects. In this regard, some studies showed that, if individuals engage in health-related behaviors on the basis of appearance-related reasoning, this can contribute to extreme and unhealthy behaviors (7), higher body image concerns (21), lower body appreciation (9, 22), and a lower self-esteem (8, 9). However, focusing more on intrinsic motivation (i.e., engaging in health-related behaviors to feel good or to be healthy) is theorized to be a way to mitigate these negative effects (23).

In this regard, studies in the area of advertising research have already investigated the effects of differently framed slogans on body-related outcomes. A recent study examining the effects of “objective” vs. “empowering” slogans in ads on Instagram found no difference with regard to the effects on body satisfaction of the participants (24). Another study focusing on the effects of objectifying slogans in comparison to sexually agentic slogans in ads also did not find any difference in terms of the weight dissatisfaction of the participants (25). However, these studies did not focus on health contents but on promoting products, such as a perfume (24) or a bra (25). Moreover, these studies focused only on young women, while men might also be negatively affected by idealized media portrayals (26). Therefore, it seems important to investigate how framing in connection to health-related behaviors affects body-related outcomes of the participants and with a more diverse sample.

To our knowledge, only one experimental study focused on the effects of framing (appearance vs. health) in connection to health-related behaviors on body-related outcomes. Aubrey (4) revealed that exposure to appearance frames in health magazine headlines leads to more body shaming and appearance-related motivation to exercise compared with exposure to health frame headlines. Once again, this study focused only on young women.

Some studies showed that when investigating the effects of media presentations on body-related outcomes, it was also important to measure effects on mood. These concepts seem to be closely connected (10, 27–29). Based on the theoretical considerations (14) and initial results obtained in this area of empirical research (4), we hypothesize the following:

*H1: Participants in the appearance frame condition will show (a) lower levels of positive mood, (b) higher levels of negative mood, and (c) lower levels of body satisfaction compared to participants in the health frame condition*.

### Effects on Body-Related Outcomes in Different Contexts

Based on the social comparison theory (30), individuals tend to compare themselves as a means of self-exploration. This social comparison also applies to media models. Thus, the media has the power to establish expectations on how individuals should look or behave (31).

Many experimental studies investigated the effects of body- or health-related social media posts (27, 32) as well as that of body- or health-related magazine content (4). These studies showed that exposure to such content can lead to negative effects on mood and body satisfaction (27, 29, 33). Again, these studies focused only on young women. Overall, cross-sectional studies (34–37), longitudinal studies (10), and reviews and meta-analyses (12, 26, 38) with more diverse samples revealed that media usage is associated with body-related concerns. But what media formats shape these body-related outcomes the most and how?

In this regard, some studies investigated whether different media formats affect body-related outcomes differently. Comparing the effects of magazine and television usage, a study showed that both media formats are associated with lower levels of body satisfaction (37). Another study revealed that social media posts more than magazine contents trigger comparison tendencies (10, 33). This might be due to the reason that social media content is associated with greater realism, as mainly “peers” present their content on these platforms (11). Furthermore, based on the social comparison theory (30), individuals are more likely to engage in comparison processes with other individuals that seem similar to themselves.

Based on this empirical support (12) and on the theoretical assumptions of the social comparison theory (30), we argue that social media posts might have a greater influence on the body-related concerns of the participants compared to magazine content (10). Therefore, in this study, even when the messages emphasize health-related aspects of health behaviors (i.e., healthy eating, doing sports), the social media context might still trigger an upward comparison (30). Prior studies showed that engaging in upward comparison was associated with a higher negative mood, a lower positive mood, and a greater body dissatisfaction (28, 39). Thus, we hypothesize the following:

*H2: Participants in the social media condition will show (a) lower levels of positive mood, (b) higher levels of negative mood, and (c) lower levels of body satisfaction compared to participants in the magazine condition*.

### Interaction of Framing and Context

Even though previous research identified that framing (4, 23) as well as the context (10, 37) can influence body-related concerns, no study so far has investigated the interaction effect on body-related outcomes using a diverse sample. Presenting appearance frames in connection with health-related topics can lead to negative effects (4); however, it is unclear whether presenting these frames in different contexts (magazine vs. social media) might sustain some of these negative effects (10, 21). However, it might be also possible that presenting appearance frames on social media even strengthen the negative effects of the frame on body-related outcomes. Since no study investigated these assumptions, we pose the following research question:

*RQ1: How will the presentation of health-related behavior on different media and using different frames influence participants' (a) positive mood, (b) negative mood, and (c) body satisfaction?*

### Interaction of Framing and Levels of the BMI

We also lack insights as to how the interaction of the frames and BMI of the individuals affect these body-related outcomes. Studies revealed that overweight and obese participants (i.e., women with a BMI above average) were prone to experience negative body-related outcomes, such as eating-, weight-, and shape-related concerns (40–42). Furthermore, across male and female genders, higher levels of BMI were associated with lower levels of body appreciation (43) and higher levels of body dissatisfaction (44).

Based on the *Social Comparison Theory* of Festinger (30), the exposure to appearance-focused framing might add to this relationship: Since Western beauty ideals concentrated on very lean physiques for the past few decades (45–47), individuals with a higher BMI might be more likely to perceive the discrepancy between the present body ideal and that of their own. Therefore, they might be more sensitive to the appearance-focused content, and thus, our presumed effects of appearance frames might be more prevalent.

To our knowledge, only two experimental studies have investigated the interaction of framing health messages and BMI of the individuals till date (4, 48). Segar et al. (48) focused on overweight and obese individuals and how different gain-frames of physical activity (i.e., frames: better health vs. weight loss vs. daily well-being) affected their body image. They found a significant interaction effect of frame and BMI, in that the frames had a greater impact on overweight women than they did on obese women.

Similar to the aim of this study, Aubrey (4) investigated the effects of appearance- and health-framed messages on body-related self-perceptions and the interaction effect it had with BMI in women. She found that, in young women who were underweight and overweight (in comparison to young women with normal weight), exposure to appearance-framed health messages increased the appearance-related motivation to exercise. She found no significant interaction effect of the frame and BMI regarding the state of self-objectification and body shame of an individual.

To our knowledge, no study has focused on the interaction effect of appearance- and health-focused framing and BMI for mood and body satisfaction. Since these variables are associated with symptoms of depression, eating disorders, and excess weight status (49, 50), investigating these variables is of the utmost importance.

Therefore, in this study, we investigated the interaction of appearance- and health-focused framing and BMI for these variables. Due to the scarce empirical support so far, we refrain from formulating a hypothesis and state the following research question instead:

*RQ2: How will participants' level of BMI influence the effects of framing on participants' (a) positive mood, (b) negative mood, and (c) body satisfaction?*

## Method

The study follows a 2 (appearance vs. health frames) ×2 (social media vs. magazine website) between-subjects experimental design. The data was collected online in Germany and in German by a globally acting professional survey provider (Dynata). We randomly assigned participants to one of four conditions: appearance frames on social media (*N* = 68), appearance frames on magazine websites (*N* = 72), health frames on social media (*N* = 69), or health frames on magazine websites (*N* = 77). For each condition, the visual content remained the same, while the textual content varied in terms of its framing (appearance cues: “good looking,” “attractive”; health cues: “feel good,” “healthy”). Depending on the condition, participants viewed three social media posts /magazine website articles' depicting a male as well as three social media posts /magazine website articles starring a female protagonist, totaling six social media posts /magazine website articles. All social media accounts and magazine websites were fictitious (see APPENDIX). The data set has been uploaded to the open science framework (OSF)^1^.

### Sample

Participants were recruited by a professional data collection company. We used a quota sample based on the distribution of age, gender, and education in Germany. Initially, 300 participants took part in the study. We excluded some cases due to extremely short- and long-response periods (*n* = 14; cut-off point: <10 min and three times longer than the mean time; *M* = 27.77, *SD* = 13.75). Our final sample consisted of *N* = 286 participants (50.0% female; 50.0% male). The age of the participants ranged between 18 and 65 years (*M* = 44.47, *SD* = 13.76). Based on the quota-distribution for Germany, 10.5% of our participants had received little education (e.g., no school graduation, primary school), 44.7% received moderate education (e.g., high school, vocational school), and 44.7% of our participants were highly educated (e.g., University degree).

### Measures

#### Dependent Variables

We used computer-based visual analog scales to measure our dependent variables both, before (=T1) and after (=T2) exposure to the experimental stimuli. Participants indicated their answers by moving a vertical marker on a labeled horizontal line (0 = *not at all* to 100 = *very much*).

##### Negative and Positive Mood

We asked participants to state how they currently feel by rating the following dimensions of mood: “depressed,” “anxious,” “confident,” and “happy.” We adopted this measure from previous studies (51). Following the theoretical approach of a bivariate view (52), we measured positive and negative mood as separate dimensions. Accordingly, we combined ratings of “happy” and “confident” to measure positive mood (T1: α = 0.76, *M* = 63.44, *SD* = 23.65; T2: α = 0.83, *M* = 63.37, *SD* = 26.06), and “depressed” and “anxious” to measure negative mood (T1: α = 0.81, *M* = 18.39, *SD* = 22.98; T2: α = 0.83, *M* = 17.42, *SD* = 22.67). We calculated the change scores for both positive (*M* = −0.07, *SD* = 11.08) as well as negative moods (*M* = −0.97, *SD* = 12.44).

##### Body Satisfaction

Based on previous research (51), we measured body satisfaction by asking “how satisfied are you are with your overall appearance?” “… your weight?” “… your body shape?” “… your build?” “… your physical attractiveness?” and “… your fitness?”. We combined them into an index measuring body satisfaction (T1: α = 0.94, *M* = 57.50, *SD* = 25.02; T2: α = 0.96, *M* = 56.48, *SD* = 27.00). Again, we calculated the change score (*M* = −1.01, *SD* = 9.14).

#### Moderator Variable

Based on the self-reported values of height and weight of the participants, we also calculated the BMI for each participant (kg/m^2^; *M* = 26.63, *SD* = 6.78; 3.2% underweight, 46.1% normal weight, 29.9% overweight, 20.8% obese). In 2016, the average BMI of the population above 18 years in Germany was 26.00 (1). Based on the fact that obesity and overweight are on the rise worldwide (2), this sample seems to present the average BMI of the population in a good way.

#### Control Variables

We controlled for the social media and magazine use of the participants using two questions based on their daily usage frequency (1 = *never*, 2 = < *10 min*, 3 = *10–30 min*, 4 = *31–60 min*, 5 = *more than 60 min*; social media use: *M* = 3.02, *SD* = 1.33; magazine use: *M* = 2.33, *SD* = 1.06).

We also controlled for gender and age of the participants.

## Results

### Manipulation Check

The appearance frames (*M* = 3.68, *SD* = 1.17) were considered more appearance focused than the health frames (*M* = 3.38; *SD* = 1.13; *t*(284) = −2.22, *p* = 0.028). Also, the health frames (*M* = 3.71, *SD* = 1.09) were considered as more health focused than the appearance frames (*M* = 3.42, *SD* = 1.17; *t*(284) = 2.18, *p* = 0.030). According to chi-square tests, 83.9% participants in the magazine website condition stated correctly that they saw a website of a magazine whereas 94.2% of participants in the social media condition stated correctly that they saw social media posts (*p* < 0.001).

### Randomization Check

We ran randomization checks for age (*F*_(3, 285)_ = 0.66, *p* = 0.580), gender (χ^2^ = 0.80, *df* = 3, *p* = 0.849), BMI (*F*_(3, 283)_ = 1.94, *p* = 0.123), social media use (*F*_(3, 285)_ = 1.62, *p* = 0.186), and magazine use (*F*_(3, 285)_ = 1.62, *p* = 0.186). Thus, randomization seems successful.

### Data Analysis

The dependent variables in the proposed moderated model were either positive mood, negative mood, or body satisfaction. The frame of the stimuli functioned as an independent variable (0 = health frame; 1 = appearance frame), while the context (0 = magazine website; 1 = social media) and the BMI of the participants acted as moderators. We ran a set of OLS-based bootstrapped resampling estimations using SPSS PROCESS 3 macro model no. 2 [10,000 bootstraps; (53)] for all the three dependent variables separately. We controlled for gender, age, social media, and magazine use of the participants. We reported the unstandardized regression coefficients (*b*).

### Hypotheses Testing

#### Dependent Variable: Positive Mood

The conditional effect of the frame on positive mood was statistically significant (*b* = −3.86, *p* = 0.036, 95% CI [−7.47, −0.25]). In line with hypothesis H1a, exposure to the appearance frame was negatively related to positive mood compared with exposure to the health frame.

Also, the conditional effect of the context on positive mood was statistically significant (*b* = −4.43, *p* = 0.016, 95% CI [−8.00, −0.85]). Exposure to social media posts was negatively related to positive mood compared with exposure to magazine articles. This result aligned with hypothesis H2a.

For RQ1a, we examined the interaction effect of the frame and context on positive mood. It was not statistically significant (*b* = 4.26, *p* = 0.106, 95% CI [−0.92, 9.44]).

**Figure 1:**
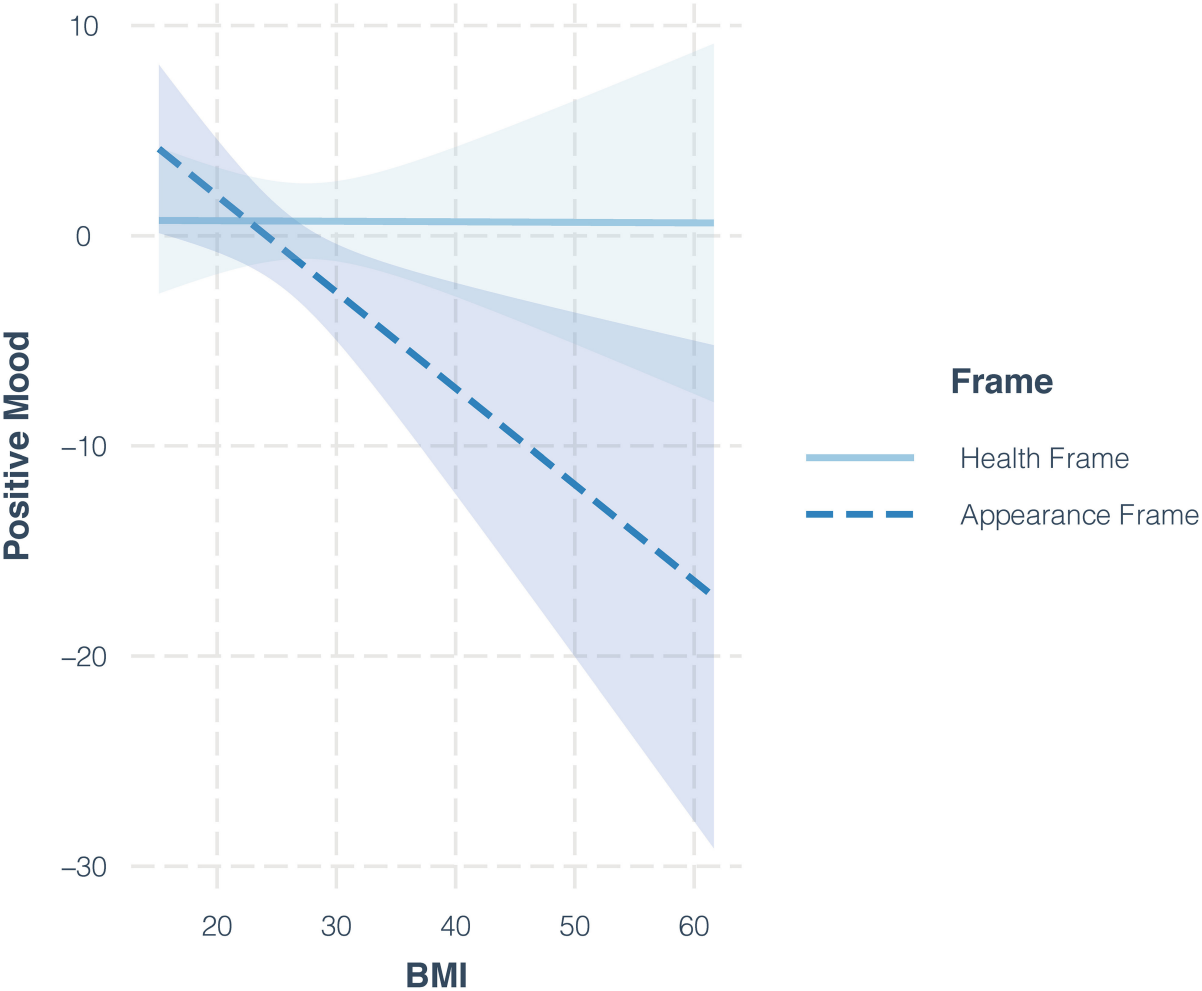
Interaction effects of the frame and level of BMI of the participants on positive mood.

For RQ2a, we investigated the interaction effect of the frame and BMI on positive mood. This interaction showed statistical significance (*b* = −0.46, *p* = 0.026, 95% CI [−0.86, −0.06]). The nature of this effect is shown in Figure 1. The result shows that the effect of the appearance frame on positive mood was negative if the BMI of the participants was high. We also conducted a Johnson–Newman test to investigate at what level this negative interaction occurs. The results revealed that, for participants with a BMI of 26.04 and above, the effect of being exposed to appearance frames on positive mood is significantly negative.

In the hypothesized moderation model control variables had no significant effect on positive mood (*ps* > 0.05; see Table 1).

**Table 1:** Moderated analysis explaining positive mood.

**Independent variables**	**Positive mood**		
	***b***	***SE***	***P-value***
**Frame (appearance)**	**−3.86**	**1.83**	**0.036**
**Medium (social media)**	**−4.43**	**1.82**	**0.016**
Social media use	−0.73	0.53	0.172
Magazine use	−0.43	0.63	0.494
Age	0.05	0.05	0.391
BMI	−0.00	0.12	0.983
Gender (female)	−1.51	1.31	0.253
Appearance frame * Social media	4.26	2.63	0.106
**Appearance frame** * **BMI**	**−0.46**	**0.20**	**0.026**

*Macro PROCESS 3, Model 2 with 10,000 bootstrap samples; N = 284; R^2^ = 0.06*.

*Effects based on linear regression models. Significant relationships are visualized bold in order to support the readability of these tables*.

#### Dependent Variable: Negative Mood

The conditional effect of the frame on negative mood (*b* = 0.06, *p* = 0.976, 95% CI [−4.04, 4.17]) as well as the conditional effect of the context on negative mood were not statistically significant (*b* = −2.44, *p* = 0.239, 95% CI [−6.50, 1.63]). Therefore, hypotheses H1b and H2b were not supported. In other words, in our hypothesized model, neither the frame nor the context affected the state of the negative mood of the individuals.

For RQ1b, we investigated the interaction effect of the frame and context on negative mood. This relationship did not show statistical significance (*b* = 4.60, *p* = 0.125, 95% CI [−1.30, 10.50]). Also, the interaction effect of the frame and BMI as investigated for RQ2b was not statistically significant (*b* = −0.24, *p* = 0.297, 95% CI [−0.70, 0.21]).

None of our control variables showed statistical significance in our hypothesized moderation model for negative mood (*ps* > 0.05; see Table 2).

**Table 2:** Moderated analysis explaining negative mood.

**Independent variables**	**Negative mood**		
	***b***	***SE***	***P-value***
Frame (appearance)	0.06	2.09	0.976
Medium (social media)	−2.44	2.07	0.239
Social media use	−0.61	0.61	0.313
Magazine use	0.46	0.71	0.518
Age	0.00	0.06	0.999
BMI	−0.09	0.14	0.540
Gender (female)	−0.03	1.50	0.987
Appearance frame * Social media	4.60	2.99	0.125
Appearance frame * BMI	−0.24	0.23	0.297

*Macro PROCESS 3, Model 2 with 10,000 bootstrap samples; N = 284; R^2^ = 0.03*.

*Effects based on linear regression models*.

#### Dependent Variable: Body Satisfaction

The conditional effect of the frame on body satisfaction was not statistically significant (*b* = 0.19, *p* = 0.899, 95% CI [−2.75, 3.12]). H1c was not supported.

However, the conditional effect of the context on body satisfaction showed statistical significance (*b* = −3.60, *p* = 0.016, 95% CI [−6.51, −0.69]). In line with H2c, exposure to social media posts resulted in lower body satisfaction in comparison to website articles.

For RQ1c, we investigated the interaction effect of the frame and the context on body satisfaction. This interaction was not statistically significant (*b* = 1.59, *p* = 0.457, 95% CI [−2.62, 5.81]). Also, as for RQ2c, we did not find a statistically significant interaction effect of the frame and BMI of the individuals on body satisfaction (*b* = 0.05, *p* = 0.749, 95% CI [−0.27, 0.38]).

In the hypothesized moderation model, among the control variables, only gender showed a statistically significant effect for body satisfaction (*b* = −3.44, *p* = 0.001, 95% CI [−5.55, −1.34]). In this regard, women reported lower body satisfaction than men.

For an overview of the hypotheses and results, see Tables 3, 4.

**Table 3:** Moderated analysis explaining body satisfaction.

**Independent variables**	**Body satisfaction**		
	***b***	***SE***	***P-value***
Frame (appearance)	0.19	1.49	0.900
**Medium (social media)**	**−3.60**	**1.48**	**0.016**
Social media use	0.08	0.43	0.850
Magazine use	−0.99	0.51	0.053
Age	0.05	0.04	0.255
BMI	−0.10	0.10	0.330
**Gender (female)**	**−3.44**	**1.07**	**0.001**
Appearance frame * Social media	1.59	2.14	0.457
Appearance frame * BMI	0.05	0.17	0.749

*Macro PROCESS 3, Model 2 with 10,000 bootstrap samples; N = 284; R^2^ = 0.08*.

*Effects based on linear regression models. Significant relationships are visualized bold in order to support the readability of these tables*.

**Table 4:** Overview of the assumptions and results.

**H1:** Participants in the appearance frame condition will show (a) lower levels of positive mood, (b) higher levels of negative mood, and (c) lower levels of body satisfaction compared to participants in the health frame condition.	(a) Supported (b) No support (c) No support
**H2:** Participants in the social media condition will show (a) lower levels of positive mood, (b) higher levels of negative mood, and (c) lower levels of body satisfaction compared to participants in the magazine condition.	(a) Supported (b) No support (c) Supported
**RQ1:** How will the presentation of health-related behavior on different media and using different frames influence participants' (a) positive mood, (b) negative mood, and (c) body satisfaction?	(a) No effect (b) No effect (c) No effect
**RQ2:** How will participants' level of BMI influence the effects of framing on participants' (a) positive mood, (b) negative mood, and (c) body satisfaction?	(a) Effect of the appearance frame on positive mood was negative if the BMI of the participants was high (b) No effect (c) No effect

## Discussion

The aim of this study was to gain more insights into how different framing strategies (i.e., appearance- vs. health-focused framing), different contexts (i.e., social media vs. magazine website), and the interaction of both influence the positive mood, negative mood, and body satisfaction of the participants. These results might aid health promoters in designing effective health messages to raise awareness about and counteract the rising overweight and obesity worldwide. For that, it is of utmost importance to understand how the general population reacts to different communication modes. After all, prior research has shown that poorly communicated health intervention messages can even harm individuals, in that undesired and unhealthy behaviors are pursued (54).

With regard to different framing strategies, our results slightly supported the Framing Theory (14) where exposure to appearance frames (in comparison to health frames) decreased the positive mood of the participants. With respect to our research question, this negative effect was apparent among overweight or obese participants. One might argue that this result reflects the general lower levels of body satisfaction in heavier individuals (43, 44). However, we accounted for this aspect through pre-post-measurements of our dependent variables as well as the calculation and analysis of change scores. Instead, we argue that, possibly, exposure to content that emphasizes the importance of appearance triggered overweight and obese participants to engage in upward comparisons. In support of this explanation, a recent study showed that women with a high actual-ideal body discrepancy showed lower body satisfaction while being exposed to thin-idealized models (24). Thus, especially overweight and obese individuals seem to be sensitive to the negative effects of appearance framing on body-related outcomes.

In contrast to our hypotheses and prior research findings (27, 29, 51), we did not find any effects of framing on negative mood or body satisfaction of the participants. However, these studies focused on visual stimuli and compared the effects of different picture-based interventions [e.g., exposure to thin-idealized vs. more realistic female bodies; (51)]. In contrast, we manipulated the textual content in this study. Our results suggest that, while textual appearance framing can decrease positive mood, negative mood (i.e., feeling depressed and/or anxious) and the body satisfaction of the participants are not affected.

Moreover, our results imply that irrespective of the frame type, the social media context (in comparison to a magazine context) can negatively impact positive mood and body satisfaction of the consumers. We interpret these findings with the theoretical foundation of the Social Comparison Theory (30): We argue that participants in the social media condition compared themselves to the fictive Instagram bloggers to a greater extent in terms of both, health- and appearance-framed contents. This assumption aligns with prior research showing that social media posts more than magazine content triggered comparison tendencies (10, 33). This might be due to the fact that Instagram bloggers are often perceived as close peers and thus, social media content is ascribed a greater reality than magazine contents (33). Thus, mere exposure to health-related social media content can lead to negative effects on positive mood and body satisfaction compared with exposure to the same contents on magazine websites. However, these theoretical and empirical implications raise the question of whether the context—and not the message itself—is the main driver for body-related outcomes.

Again, we did not find any effects on negative mood. Thus, exposure to health-related content on different media channels does not influence the negative mood (i.e., feeling depressed and/or anxious) of the participants. The reason might be that negative mood, as operationalized in our study, refer to the severe psychological states such as anxiety and depression. It seems reasonable that exposure to social media posts does not make respondents more depressive or anxious, even after, for instance, social upward comparisons. However, positive moods may be more easily influenced by social media posts compared to negative ones. This finding suggests that a decline in positive mood does not automatically correspond to an increase in negative mood, thus signaling the necessity to operationalize both in empirical research.

Another aim of this study was to investigate the interaction effects between the frame and the context. However, our results did not support any interaction effect of the two with regard to our dependent variables. Thus, the negative effects of appearance framing and the social media context did not cumulate nor did they mitigate each other.

In this study, we kept the images as neutral as possible and only varied the framing of the textual content and the contexts. Nevertheless, the pictures showed male and female protagonists who might be perceived as thin-ideal. According to the WHO (1), our sample can be classified as slightly overweight. Therefore, our participants might have observed some discrepancy between the depicted body types and their own, which might have resulted in greater upward comparison, especially in the social media condition due to higher perceived realism (11). According to literature, upward comparison is associated with lower levels of positive mood, higher levels of negative mood, and greater body dissatisfaction (28, 39). Our results align with these findings to a certain degree. Furthermore, complementing this interpretation, Hendrickse et al. (24) showed that exposure to thin-idealized models can lead to higher body-related concerns regardless of the presented textual message. It might be the case that, in our study, participants perceived the depicted protagonists as thin-ideal as they appeared in the promotion of health behaviors (i.e., healthy eating, doing sports). Since promoters of health behaviors usually represent the thin-ideal (47), this inference seems plausible. However, the interpretation remains an assumption only because we did not measure the perceptions of the participants for the depicted body types nor the perceived discrepancy between their own bodies and the depicted ones. These aspects should be considered by future research.

In summary, our findings reveal that the context—among many others—is one important parameter that needs to be more carefully considered by (1) scholars investigating media effects on body-related outcomes as well as (2) health advocates when promoting health-related behaviors. Health promoters need to be very clear when promoting health-related behaviors. Therefore, health promotion should try to focus on the advantages for the overall health and well-being of an individual and should omit putative appearance benefits. Furthermore, our results suggest that social media, although appealing at the first glance in terms of potential reach and cost efficiency, might come with certain disadvantages for promoting good nutrition and physical exercise. In this study, exposure to the social media context resulted in reduced positive mood and body satisfaction, indicating that the context of social media (i.e., Instagram in this case) is already connoted. Thus, health campaigners should be wary and must pay attention to the influential role of media context when promoting health behaviors. Sometimes coming back to more traditional media, such as magazines, might be even more beneficial.

Our study also contributes to the scholarly debate by investigating the effects with a more diverse sample. Previous studies primarily focused on the effects in young and highly educated women (12). However, studies showed that a broader population is affected by media effects on body-related outcomes, irrespective of age and gender (13). Moreover, social media consumption is on the rise in all age groups and genders (55). Therefore, the strong focus on young women in the research of media effects and body-related outcomes is neither commensurate nor timely. This study only represents a small yet important step onto the right path of investigating and understanding media effects and body-related outcomes for the broader population.

### Limitations and Further Research

As every study, this study also has its limitations. First of all, as already mentioned, we aimed to present neutral pictures. However, participants might have perceived the depicted individuals as thin-idealized models. As in this context, a recent study showed that visual stimuli are more salient than textual messages (24). Our participants might have paid more attention to the pictures and lesser attention to the messages which we manipulated. This might explain some of the null findings of the appearance framing and also those regarding the interaction effects. Thus, future studies should measure the perceptions of the participants for the displayed body types and consider visual stimuli without any body presentation.

Second, body-related outcomes seem to be closely related to comparison behavior in the context of this study. Future research should focus further in this area. This seems especially important with regard to social media contexts due to the higher degrees of perceived realism of its contents (11). Specifically, research that builds on Social Comparison Theory of Festinger (30) should pay attention to the comparison tendencies of the participants (10), their actual-ideal body discrepancy (24), and the perceived discrepancy between the presented bodies and their own.

Third, based on previous studies in this area of research (24, 51), we measured the positive mood, negative mood, and body satisfaction of the participants using a pre-post-measure. This might have triggered the heightened awareness of the participants for the following stimuli. We still argue that, in order to draw meaningful conclusions about the decrease or increase of body-related outcomes, pre-post-measures are essential. However, future research should plan more time between the pre- and the post-measurement of body-related outcomes. This might help to minimize priming effects. Fourth, as was the case in other studies [e.g., (4, 41, 48)], we measured the BMI of participants based on self-reports. Of course, this measurement comes with limitations in terms of downward biases when relying on self-reported levels of BMI of the individuals (56). This needs to be considered when drawing inferences from our results on the interaction effect of appearance framing and BMI.

Lastly, this study solely investigated the short-term effects. Thus, we are not able to draw any inferences on the long-term effects or repeated exposure to appearance framing in different contexts. Further research should try to tackle this research gap.

## Conclusion

This was one of the first studies to investigate the effects of health vs. appearance framing in different contexts. This is important because these strategies are widely used in social media (3, 6) as well as in magazines (5, 17). However, emphasizing the goal of improving the appearance of an individual rather than contributing to the individual's health can lead to certain negative effects. Our results imply that, in the promotion of health behaviors, *how* a message is communicated and, more importantly, the *context* in which it is communicated matter. Therefore, appearance framing and health framing, both, can lead to negative effects when presented in a social media context compared to presenting the same content on magazine websites. Health promoters need to be aware that not only the manner in which a message is conveyed but also the context in which it appears can lead to different and partially undesired effects. Thus, the content as well as the context should be taken into consideration when designing health messages. Since these effects occurred irrespective of the gender or age of the participants, studies in this area of research should also set their focus on more diverse samples.

## Data Availability Statement

The raw data supporting the conclusions of this article will be made available by the authors, without undue reservation.

## Ethics Statement

Ethical review and approval was not required for the study on human participants in accordance with the local legislation and institutional requirements. The patients/participants provided their written informed consent to participate in this study.

## Author Contributions

AB and IS conceptualized the study, the data collection instruments, collected data, and did the initial analysis. AB and SN finalized the analysis and drafted the initial manuscript and reviewed and revised the manuscript. JM supervised data collection instruments as well as the data collection, and reviewed and revised the manuscript. All authors approved the final manuscript as submitted and agree to be accountable for all aspects of the work.

## Conflict of Interest

The authors declare that the research was conducted in the absence of any commercial or financial relationships that could be construed as a potential conflict of interest.

## Publisher's Note

All claims expressed in this article are solely those of the authors and do not necessarily represent those of their affiliated organizations, or those of the publisher, the editors and the reviewers. Any product that may be evaluated in this article, or claim that may be made by its manufacturer, is not guaranteed or endorsed by the publisher.

## Footnotes

^1^For the data set follow this link: https://osf.io/7fw5q/?view_only=03cd1149a55a4bfd8e3120afb8122884.
